# Nrf2 stabilization prevents critical oxidative damage in Down syndrome cells

**DOI:** 10.1111/acel.12812

**Published:** 2018-07-20

**Authors:** Emiliano Zamponi, Nahuel Zamponi, Pinar Coskun, Gonzalo Quassollo, Alfredo Lorenzo, Sergio A. Cannas, Gustavo Pigino, Dante R. Chialvo, Katheleen Gardiner, Jorge Busciglio, Pablo Helguera

**Affiliations:** ^1^ Instituto de Investigación Médica Mercedes y Martín Ferreyra INIMEC‐CONICET‐Universidad Nacional de Córdoba Cordoba Argentina; ^2^ Department of Medicine, Division of Hematology and Medical Oncology Weill Cornell Medicine New York New York; ^3^ Department of Neurobiology and Behavior, Institute for Memory Impairments and Neurological Disorders and Center for the Neurobiology of Learning and Memory University of California Irvine Irvine California; ^4^ Instituto de Física Enrique Gaviola (IFEG‐CONICET) FAMAFyC, UNC Cordoba Argentina; ^5^ Center for Complex Systems and Brain Sciences (CEMSC3) UNSAM San Martin Argentina; ^6^ Department of Pediatrics, Linda Crnic Institute for Down Syndrome University of Colorado Denver School of Medicine Aurora Colorado

**Keywords:** Down syndrome, Dp16, mitochondrial catalase, Nrf2, oxidative stress, PKCδ

## Abstract

Mounting evidence implicates chronic oxidative stress as a critical driver of the aging process. Down syndrome (DS) is characterized by a complex phenotype, including early senescence. DS cells display increased levels of reactive oxygen species (ROS) and mitochondrial structural and metabolic dysfunction, which are counterbalanced by sustained Nrf2‐mediated transcription of cellular antioxidant response elements (ARE). Here, we show that caspase 3/PKCδdependent activation of the Nrf2 pathway in DS and Dp16 (a mouse model of DS) cells is necessary to protect against chronic oxidative damage and to preserve cellular functionality. Mitochondria‐targeted catalase (mCAT) significantly reduced oxidative stress, restored mitochondrial structure and function, normalized replicative and wound healing capacity, and rendered the Nrf2‐mediated antioxidant response dispensable. These results highlight the critical role of Nrf2/ARE in the maintenance of DS cell homeostasis and validate mitochondrial‐specific interventions as a key aspect of antioxidant and antiaging therapies.

## 1 INTRODUCTION

Down syndrome (DS), caused by the presence of an extra copy of human chromosome 21 (Hsa21), is characterized by a complex clinical phenotype that includes developmental alterations, intellectual disability and accelerated aging (Roizen & Patterson, 2003). An expanding number of studies have reported the presence of chronic oxidative stress and mitochondrial dysfunction in DS at both the cellular and organismal levels (Annerén & Epstein, 1987; Busciglio & Yankner, 1995; Antonarakis et al., 2004; Lott & Dierssen, 2012; Campos et al., 2011). Recently, we reported that decreased mitochondrial activity, increased ROS and mitochondrial fragmentation in DS cells are associated with transcriptional activation of the nuclear factor (erythroid‐derived 2)‐like 2 (Nrf2) signaling pathway (Helguera et al., 2013). Nrf2, a master regulator of antioxidant response elements (ARE), is a cytoplasmic protein constitutively synthesized and rapidly degraded after ubiquination mediated by its repressor, Kelch like‐ECH‐associated protein 1 (Keap1)(Itoh et al., 1999; Nguyen et al., 2009; Zhang et al., 2004). Under oxidative conditions, the cysteine‐rich interacting domain in Keap1 changes its conformation, disabling its default association with Nrf2. As a result, Nrf2 accumulates in the cytoplasm and after phosphorylation by PKCδ, pNrf2ser40 translocates into the nucleus where it activates transcription of antioxidant response elements (ARE) (Niture et al., 2009; Villeneuve et al., 2010; Zhang & Hannink, 2003).

The “Free Radical Theory of Aging” (Harman, 1956) postulates a link between the progressive accumulation of macromolecular damage caused by reactive oxygen species (ROS) and the aging process, particularly cell arrest in mitotic cells, referred to as cellular senescence. The main source of ROS generation inside cells are electrons released from the mitochondrial electron transport chain that reduce molecular oxygen to form superoxide anions (O_2_^•−^), which in turn are converted to hydrogen peroxide (H_2_O_2_) that generate reactive hydroxyl radicals (^•^OH) unless H_2_O_2_ is inactivated by glutathione peroxidase or peroxisomal catalase. The impact of ROS accumulation in mammalian lifespan was directly tested in a transgenic mouse overexpressing human catalase with a mitochondrial targeting sequence (mCAT) (Schriner, 2005). mCAT expression resulted in increased longevity without interference on major transduction pathways. Life span extension in mCAT mice was associated with reduced cardiac pathology, decreased DNA oxidative damage, protection against age‐related insulin resistance, and improved proteostasis, underscoring the role of cumulative oxidative damage in aging associated pathologies (Lee et al., 2010; Mao et al., 2012; Treuting et al., 2008; Umanskaya et al., 2014). To further understand the role of Nrf2 stabilization in homeostasis and survival of trisomic cells, we assessed the Nrf2 signaling pathway as well as the effect of mCAT in DS human fibroblasts (HF), and mouse embryonic fibroblasts (MEF) derived from Dp16 mice, a segmentally trisomic model that reproduces several clinical phenotypes present in people with DS, including cognitive impairment (Belichenko et al., 2015; Li et al., 2007; Yu et al., 2010). We found that chronic canonical Nrf2 activation is required to prevent extensive oxidative damage and is critical for survival of both DS HF and Dp16 MEF. mCAT expression reverted mitochondrial dysfunction and reduced ROS levels leading to the inactivation of Nrf2 stabilization and translocation. Thus, Nrf2 is required in trisomic cells to maintain viability and cellular function. However, its activity can be rendered dispensable by an effective mitochondrial‐targeted intervention.

## 2 RESULTS

### 2.1 Oxidative stress and mitochondrial dysfunction in DS HF and Dp16 MEF

**Figure 1:**
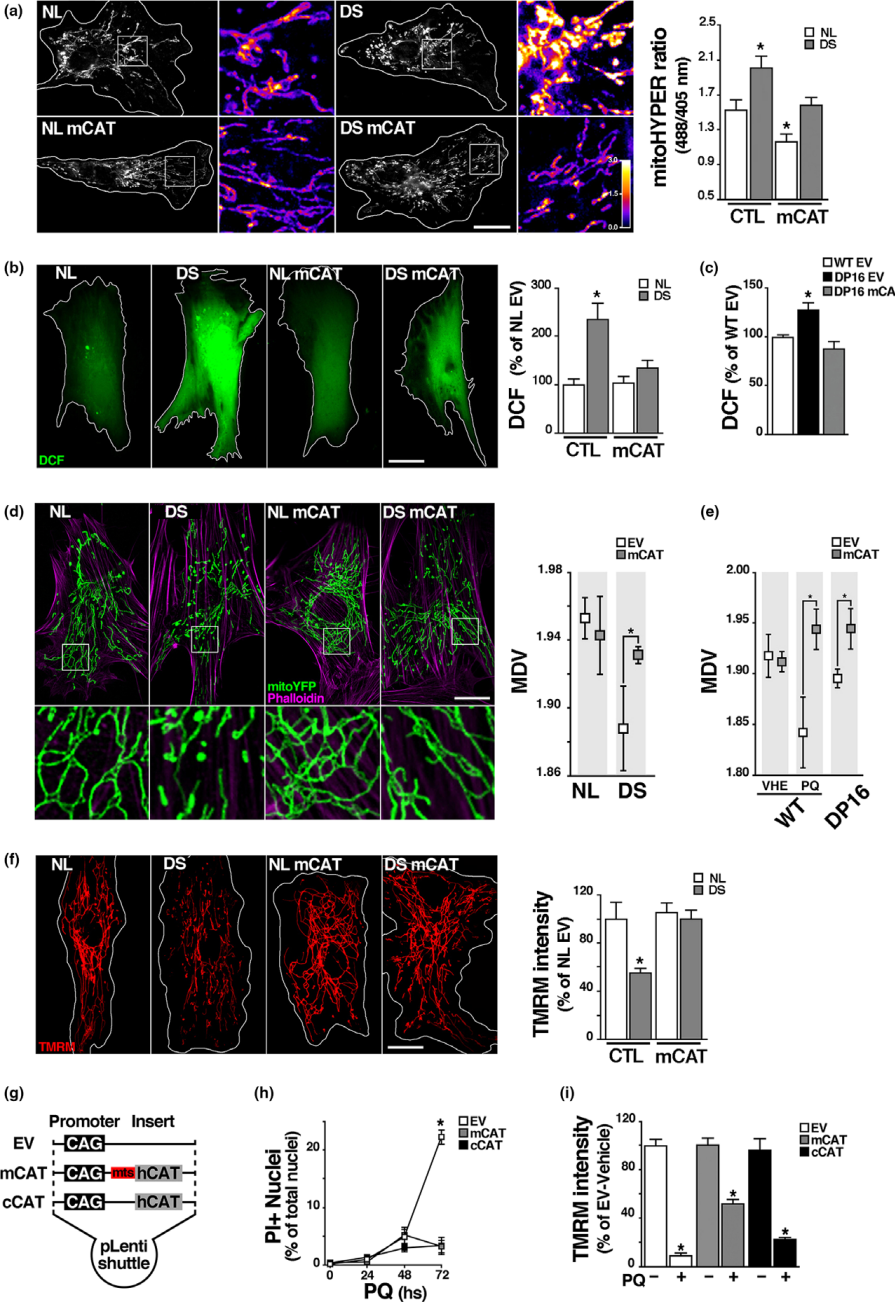
Oxidative stress and mitochondrial dysfunction in DS HF and Dp16 MEF and antioxidant and cytoprotective effects of catalase. (a) Quantification of mitochondrial H_2_O_2_ in NL and DS HF using the mitoHyPer radiometric sensor. Cultures expressing mCAT and nonexpressing controls were transfected with mitoHyPer for 48 hr and then fixed. ROS concentration was calculated as the ratio between 488 and 405 nm fluorescence values for each pixel. Heat bar: Yellow and blue indicate highest and lowest ROS levels respectively. Quantification of the ratio between fluorescence intensity at 488 and 405 nm shows a marked increase in DS cells, which is prevented by mCAT expression. Heat bar: Yellow indicates highest and blue lowest ROS levels. * *p *< 0.05. Scale bar = 20 μm. (b) Total ROS levels were measured using DCF in native and transduced NL and DS HF (NL, DS, NL mCAT and DS mCAT). Quantification shows a significant increase in DCF levels in DS, which is prevented by mCAT (DS mCAT). * *p *< 0.05. Scale bar = 20 μm. (c) Quantification of DCF intensity as a measure of total cellular ROS show a increase on DS mouse model Dp16 MEF (Dp16) compared to control (WT EV), which is prevented in cells expressing mCAT (Dp16 mCAT). * *p *< 0.05. (d) Mitochondrial network integrity was analyzed in NL and DS HF coexpressing mitochondrial targeted YFP (mitoYFP); in the same set of conditions described above, using nodes mean degree value (MDV) as parameter of morphological integrity (see methods). Alexa‐546 phalloidin was used to highlight cell boundaries. Significant fragmentation in DS mitochondrial network is recovered by mCAT. *p < 0.05. Scale bar = 20 μm. (e) Mitochondrial network quantification analysis in WT and Dp16 MEF cotransduced with EV or mCAT and mitoYFP. WT EV and WT mCAT cultures were treated with 200 mM PQ for 48 hr to induce mitochondrial stress, as positive control. MDV values showed more fragmented network structure in Dp16 and in PQ treated WT cells, that were recovered by mCAT. *p < 0.05. (f) MMP was measured using TMRM as described in Methods in NL and DS HF (NL, DS, NL mCAT, and DS mCAT). mCAT expression significantly increased MMP in DS cells. Scale bar = 20 μm. (g) Lentiviral plasmids scheme showing the promoter (CAG) and the human catalase sequence (hcat) in cytoplasmic catalase (cCAT) vector, or hcat attached to a mitochondrial targeting sequence (mts) in the mCAT construct. Control empty vector (EV) lacks coding sequences. (h) Oxidative stress‐induced cell death was assayed using propidium iodide (PI). Cultures expressing EV, mCAT or cCAT were treated with 5 mM PQ or vehicle for 0, 24, 48 and 72 hr and then incubated with 1.5 μM PI. Cell death was quantified as the ratio of PI‐positive nuclei over total nuclei stained with DAPI. (i) Mitochondrial membrane potential (MMP) analyzed with TMRM probe. Cells expressing EV, mCAT or cCAT were treated 48 hr with 5 mM PQ or vehicle; then incubated with TMRM and measured by live‐imaging. There is a significant recovery of MMP in PQ‐treated cultures expressing mCAT. Scale bar = 10 μm. * *p *< 0.05. a, b, c, d, e, and f: For each experimental condition a total of 20–30 cells were included in the analysis; h and i: six culture replicates (four 10× fields each) analyzed per condition

We recently reported chronic downregulation of mitochondrial activity in DS cells as an adaptive response to minimize oxidative damage and preserve basic cellular functions (Busciglio et al., 2002; Helguera et al., 2013). We further examined mitochondrial function and structure in DS HF and Dp16 MEF. As the mitochondrial network is the main source of cellular ROS (Balaban et al., 2005) mitochondrial ROS were quantified with a radiometric biosensor (mitoHyPer) that detects peroxide intermediates in the organelle (see methods). Cellular ROS, mitochondrial membrane potential (MMP) and network integrity were also assessed to establish the putative correlation between ROS levels and mitochondrial deficits. Consistent with our previous findings, DS cells exhibited both significantly higher mitoHyPer fluorescence ratio (Figure 1a), and increased dichlorofluorescein (DCF) signal (Figure 1b), indicative of oxidative stress. Increased DCF was also evident in Dp16 cells (Figure 1c, Supporting Information Figure S4C). Mitochondria are organized as dynamic networks of interconnected branched tubules of different lengths and shape, reflecting the organelle functionality (Friedman & Nunnari, 2014). As chronic oxidative stress in trisomic cells results in morphological changes in the mitochondrial network (Helguera et al., 2013), we asked whether structural changes could be tracked in DS HF and Dp16 MEF. Mitochondria were visualized using mitochondria‐targeted yellow fluorescent protein (mitoYFP). To analyze structural variations in mitochondrial networks we applied a recently developed computational tool that establishes a correlation between the level of fragmentation/branching and the mean degree of connectivity at every point of the network, referred as mean degree value (MDV; Zamponi et al., 2018; see methods; Supporting Information Figure S3). A fragmented mitochondrial network was detected in DS HF (Figure 1d, insets) as illustrated by a lower MDV compared to control diploid human fibroblasts (NL) (Figure 1d), and also in Dp16 MEF (Figure 1e; Supporting Information Figure S4D), albeit to a lesser extent. Wild‐type (WT) MEF treated with 200 μM paraquat (PQ) (Lang et al., 2010; Magnone et al., 2014) for 48 hr were included in the analysis as a positive control of network fragmentation. Consistent with the alterations described above, the MMP was decreased in DS cells (Figure 1f).

To evaluate the contribution of mitochondrial oxidative stress to abnormal cellular function and the effect of a mitochondrial‐targeted intervention in trisomic cells, we transduced mCAT using a lentiviral system (pLV‐eGFP; (Enomoto et al. 2013); see also methods). CAT expression vectors were designed either with a complex IV subunit‐targeting sequence to direct the protein to the mitochondrial matrix (mCAT), or without targeting sequence for cytoplasmic expression (cCAT; Figure 1g). Both constructs rendered a protein of correct molecular weight, displayed enzymatic activity, and compartmentalized correctly, as observed by immunocytochemistry and western blot of cytoplasmic‐ and mitochondrial‐enriched fractions (Supporting Information Figure S1A–D). The ability of mCAT and cCAT to protect against chronic mitochondrial oxidative stress was assayed by treating mCAT‐ or cCAT‐expressing HEK cells with 5 mM PQ for 48 hr (Supporting Information Figure S2B). PQ diverts electrons out from Complex I of the electron transport chain generating ROS in the mitochondrial compartment (Cochemé & Murphy, 2008). Both CAT constructs prevented PQ‐induced cell death (Figure 1h) and diminished oxidative damage (Supporting Information Figure S2). However, mCAT was effective in recovering MMP (Figure 1i, Supporting Information Figure S1F), and provided better protection against mitochondrial ROS (Supporting Information Figure S1E). mCAT was also appropriately transduced in DS HF, minimizing ROS accumulation, restoring mitochondrial structure and significantly improving MMP (Figures 1 and 3a). A similar mCAT antioxidant protective effect was observed when expressed in Dp16 MEF (Supporting Information Figure S4A–C).

### 2.2 mCAT normalizes proliferation and wound healing capacity of DS HF

**Figure 2:**
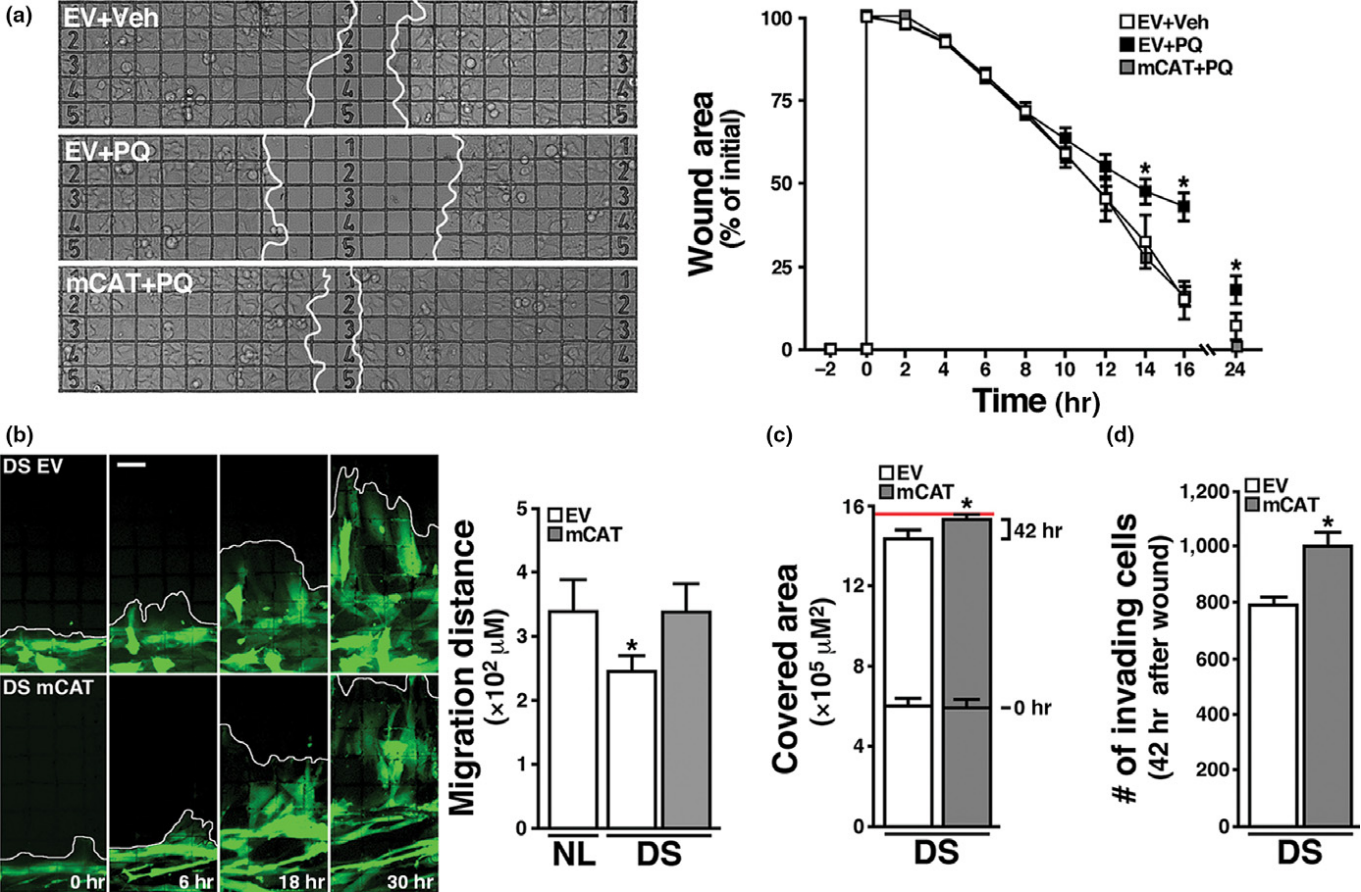
Restoration of cellular functional capacity by mCAT. (a) HEK293 cells expressing EV or mCAT where seeded and grown on a gridded glass bottom dish until confluence was reached, treated with 5 mM PQ or vehicle and subjected to a wound healing assay (see Methods). The quantification of cell‐free area over time (0, 2, 4, 6, 8, 10, 12, 14, 16 and 24 hr) indicates the cells' competence to repopulate the cleared area. mCAT markedly reduces PQ‐induced delay in wound healing in HEK293 cells. Scale bar: 100 μm. (b) DS fibroblast were co‐transduced with either EV or mCAT and a plasmid encoding cytoplasmic eYFP, seeded on a grid‐containing coverslip and subjected to wound healing assay. Images were taken 0, 6, 18 and 30 hr after injury. Scale bar = 50 μm. Chart: Quantification shows the migration distance of NL EV, DS EV and DS mCAT fibroblasts 30 hr after injury. *p < 0.05. (c) Cell‐covered area was quantified in DS cultures expressing EV or mCAT at 0 and 42 hr after injury. The red line indicates the surface of the grid area. (d) The number of cells within the grid at 42 hr was quantified using DAPI staining. *p *< *0.05. a, b, c and d: at least three IVIDI gridded glass bottom dishes, with four independent quadrants each, were processed for each experimental condition

Previous work showed that multiple cellular functions are sensitive to mitochondrial failure in DS cells (Coskun et al., 2016; Helguera et al., 2013). To assess the potential functional recovery afforded by mCAT, we analyzed cellular proliferation and motility in a wound‐healing assay. First, we asked whether ROS‐associated mitochondrial dysfunction interferes with cell repopulation after in vitro injury and, if so, whether mCAT expression can prevent it. We checked wound healing sensitivity to oxidative stress in HEK cells by injuring confluent cultures and treating them with 5 mM PQ. Wound area closure was visualized by time‐lapse microscopy as described in the methods section. PQ delayed the wound healing process, which was significant 14–16 hr postwound. These functional deficits were prevented by mCAT expression (Figure 2a).

We then examined wound healing in DS HF expressing eYFP to facilitate live cell imaging. A delay in wound closure was evident in DS cultures when compared to NL cells 30 hr postwound (Figure 2b). Quantification of the number of cells repopulating the wound gap 42 hr postwound showed a reduction in DS HF covered area and density (Figure 2c,d), while mCAT expression in these cells significantly improved all three parameters (Figure 2b–d).

### 2.3 Canonical Nrf2 stabilization via PKCδ and caspase 3 is critical for maintaining DS cellular function

**Figure 3:**
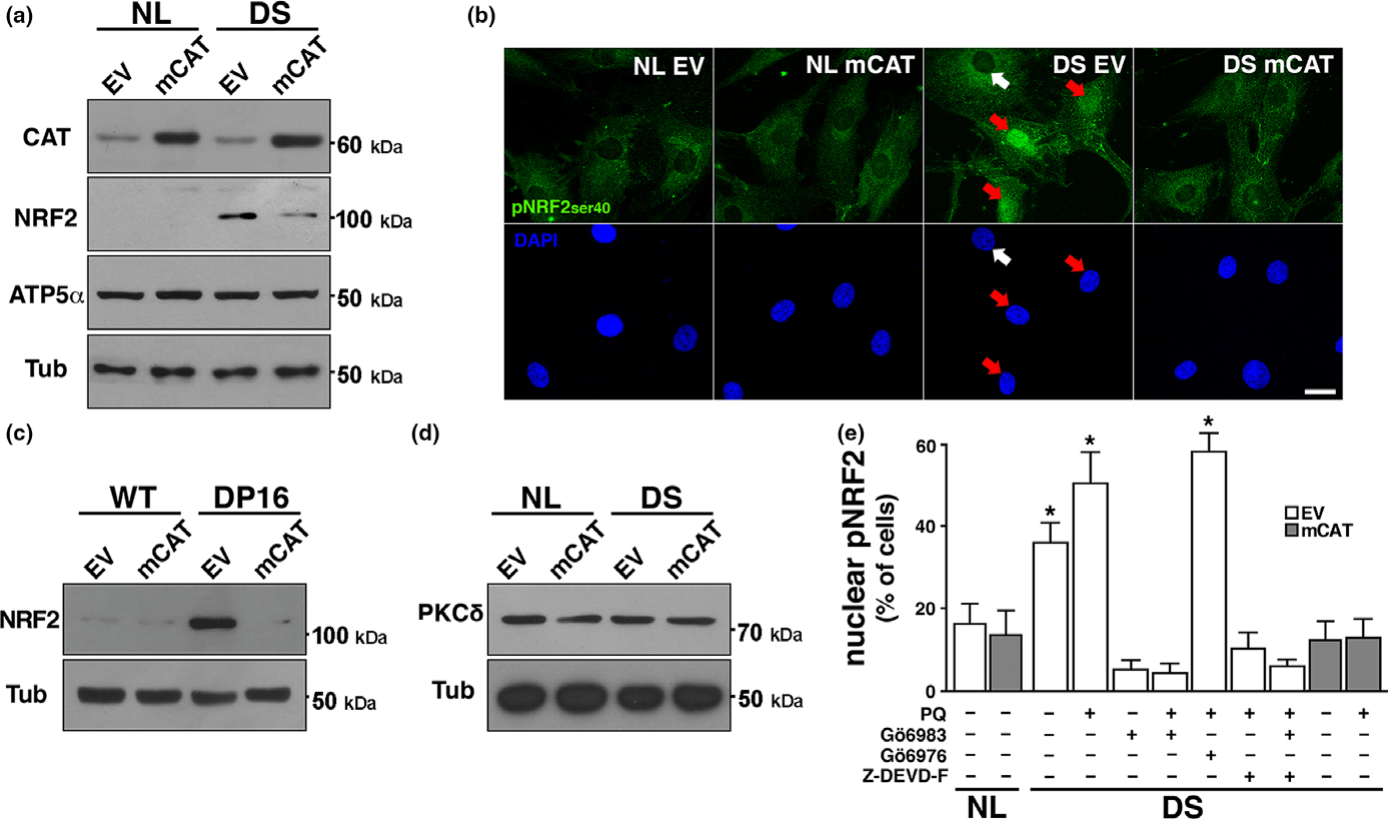
Activation of the Nrf2 signaling pathway in DS and Dp16 cells. (a) WB analysis of total NRF2 levels in NL and DS cultures infected with EV or mCAT. Samples were probed against total NRF2 and CAT. ATP5α and Tub protein levels were used as loading controls. (b) Immunofluorescence against active pNRF2ser40 (green) performed in NL and DS cultures expressing EV or mCAT. Nuclei were stained with DAPI. pNRF2 nuclear localization were considered positive when nuclear over cytoplasmic fluorescence intensity ratio was greater than 1. White arrow: no translocation; Red arrowheads: NRF2‐positive nuclei. Scale bar = 20 μm. (c) Total NRF2 levels were visualized by western blot of WT and DP16 cultures expressing EV or mCAT. Note the marked reduction of Nrf2 levels in Dp16 fibroblasts expressing mCAT. (d) PKCδ protein levels by wb in NL and DS fibroblasts infected with EV or mCAT. PKCδ expression did not change on analyzed samples. (e) Increased pNRF2 nuclear translocation in DS cells. PKCδ is activated by caspase 3, and then phosphorylates Nrf2. PKCδ regulation of NRF2 activity was determined by PKCδ inhibitor Gö6983 (2 nM). Gö6976 (2 nM), a specific inhibitor of PKCα and PKCβ, did not affect Nrf2 translocation. Casp3 dependent PKCδ activation was tested by Z‐DEVD‐FMK (Z‐DEVD‐F) Casp3 inhibitor (2 nM). DS cultures expressing EV or mCAT were treated with 200 μM PQ for 48 hr to further increase pNRFser40 nuclear localization and then incubated for 12 hr with inhibitors. *p < 0.05. b and e: 50–60 cells were included in the analysis, for each experimental condition

**Figure 4:**
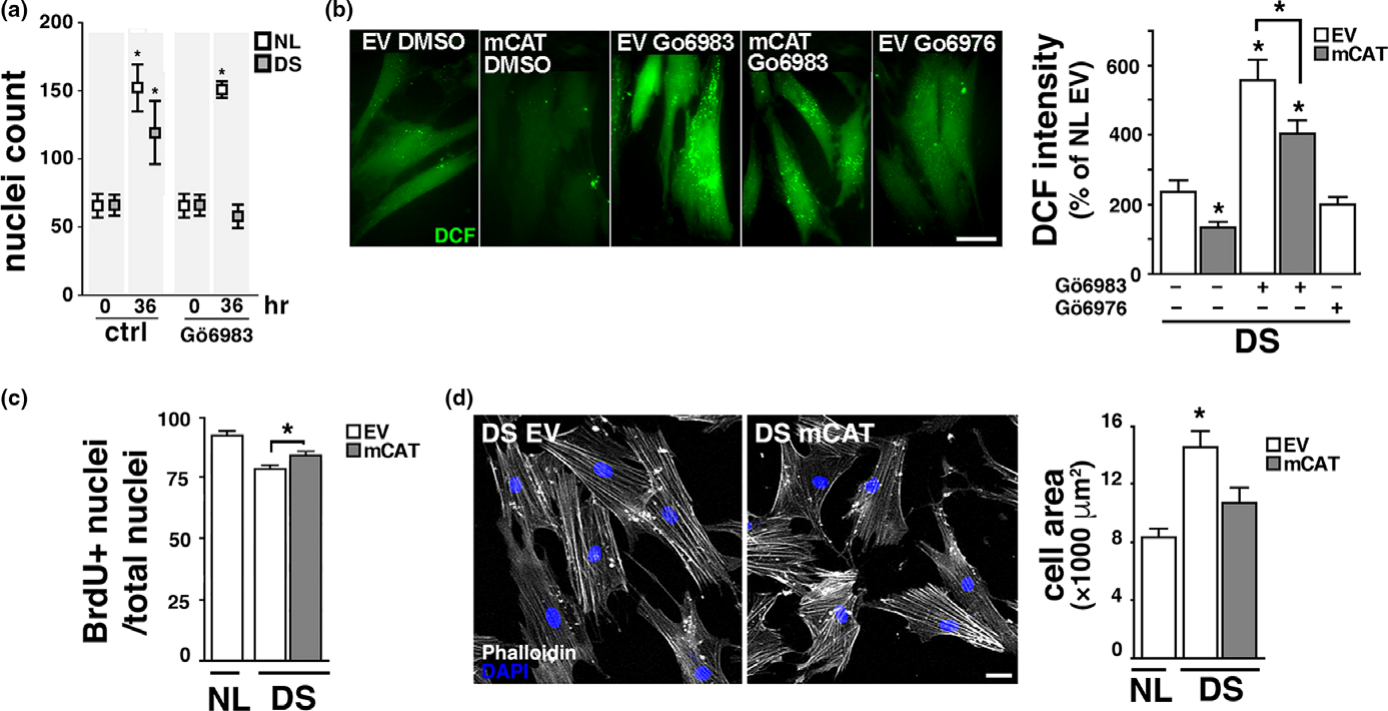
Nrf2 prevents critical oxidative damage in DS cells. (a) Inhibition of pNRF2 translocation decreases DS cell proliferation. NL and DS cells were treated with 2 nM Gö6983 or vehicle for 36 hr and then nuclei were counted. (b) Inhibition of pNRF2 translocation increases ROS generation. DS cultures expressing EV or mCAT were treated with 2 nM Gö6983 or vehicle for 12 hr and stained with DCF to measure ROS levels. Cells treated with 2 nM Gö6976 were included as a control. Scale bar = 20 μm. *p < 0.05. (c) Proliferation was evaluated by BrdU incorporation in NL or DS cultures expressing EV or mCAT. Proliferating cells (BrdU +) over total number of cells (DAPI +). *p < 0.05. (d) Increased cell area in DS fibroblasts. NL and DS cells expressing EV or mCAT vector were seeded at low density, grown for 24 hr, fixed and stained with Alexa‐546 Phalloidin and DAPI to visualize cellular an nuclear areas, respectively. Scale bar = 20 μm. * *p* < 0.05. a and c: 6 culture replicates (four 10× fields each) analyzed per condition; b: at least 20 cells included in the analysis per condition; d: 75–80 cells per condition

**Figure 5:**
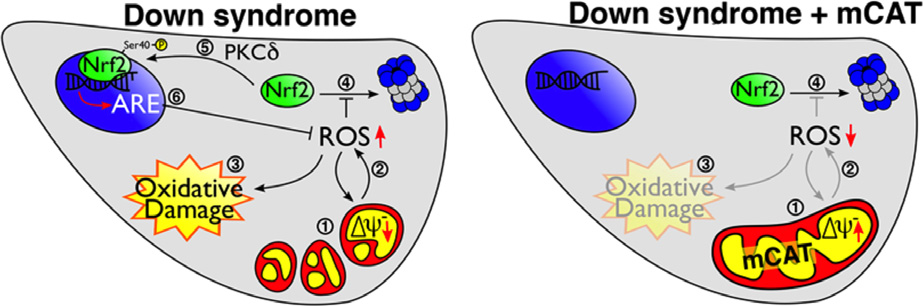
Nrf2 activation is required for DS cell survival. mCAT restores mitochondrial function and suppresses the Nrf2‐mediated antioxidant response. LEFT PANEL: In DS cells**,** mitochondrial fragmentation and low MMP (1) are caused by increased ROS production (2) leading to oxidative damage (3). Under oxidative stress conditions, proteosomal degradation of Nrf2 is inhibited (4) resulting in increased accumulation of cytoplasmic Nrf2 that, upon PKCδ phosphorylation at serine 40 (5), translocates to the nucleus and activates the expression of Antioxidant Response Elements (ARE)(6) required for DS cell survival. RIGHT PANEL: In DS cells + mCAT, catalase expression/activity (1) is sufficient to revert mitochondrial network fragmentation and MMP deficits, reducing ROS (2), minimizing oxidative damage (3), and leading to Nrf2 proteasomal degradation (4) and inactivation of the now dispensable antioxidant response

Transcriptome analysis showed an Nrf2‐associated antioxidant response in DS cells (Helguera et al., 2013). Consistent with the notion that accumulation of Nrf2 is indicative of cells under stress adjusting to neutralize oxidative damage (Niture et al., 2014; Zhang et al., 2004), we found a marked increase in Nrf2 protein levels in DS HF and Dp16 MEF cellular homogenates (Figure 3a,c) (probed with SC722 antibody, see Methods, Supporting Information Figures S5A–C and S7B,C) and strong immunofluorescent signal for pNrf2ser40 (pNrf2) in the nuclei of DS cells (see Methods, Figure 3b; Supporting Information Figures S6A and S7D,E), consistent with the presence of chronic oxidative stress. Transcriptional upregulation of Nrf2‐dependent antioxidant response elements (ARE) was confirmed by qPCR (Supporting Information Figure S8). PKCδ levels were similar in NL and DS cells (Figure 3d). To evaluate whether Nrf2 phosphorylation in DS cells is dependent on PKCδ kinase activity (Niture et al., 2009), we used the PKCδ inhibitor Gö6983 and also Gö6976, an inhibitor that do not affect PKCδ but interfere PKCα and PKCβ isoforms instead (Gschwendt et al., 1996; Martiny‐Baron et al., 1993; Stempka et al., 1999). Treatment with Gö6983 prevented pNrf2 nuclear translocation in both basal conditions and after PQ treatment, while Gö6976 had no effect (Figure 3e). As PKCδ can be activated by caspase 3 (Casp3) cleavage (Anantharam et al., 2002), we incubated PQ‐treated DS HF with the Casp3‐specific inhibitor Z‐DEVD‐FMK for 12 hr, preventing Nrf2 nuclear translocation (Figure 3e). To establish the relevance of Nrf2 activation for DS cell function, we measured oxidative damage after Gö6983 inhibition of Nrf2 phoshporylation and nuclear translocation. Under these conditions, DS HF proliferation decreased, associated with a significant burst in cellular ROS, (Figure 4a,b). mCAT significantly reduced Nrf2 levels in both DS HF and Dp16 MEF (Figure 3a,c), as well as its phosphorylation and nuclear translocation (Figure 3b,d). Furthermore, mCAT reversed Nrf2 nuclear translocation in DS HF treated with 200 μM PQ for 24 hr, where the additive effects of trisomy and PQ resulted in a dramatic increase in nuclear pNrf2 levels (Figure 3e). Not surprisingly, other antioxidants targeted to mitochondria, specifically MitoQ (1 μM) and MitoTEMPO (20 μM) also induced Nrf2 deactivation/degradation in DS HF (Supporting Information Figure S5D). Finally, we tested whether an antioxidant intervention (mCAT) would ameliorate the senescent phenotype present in DS HF, including increased cell size, a well‐characterized early marker of cellular senescence (Hayflick, 1965; Phillip et al., 2017; Rodier & Campisi, 2011). DS HF displayed significantly reduced proliferation (Figure 4c) and larger average cell area (Figure 4d). Both parameters were partially reversed by mCAT expression, restoring the proliferative capacity of DS cells. Together, as summarized in Figure 5, the above results indicate that Nrf2 nuclear translocation in DS is mediated by casp3‐activated PKCδ phosphorylation, which is critical to maintain cell homeostasis (Figure 5, left panel). mCAT expression reduced oxidative stress in DS HF, leading to a recovery in cellular metabolism and to the inactivation of Nrf2 stabilization (Figure 5, right panel).

## 3 DISCUSSION

As postulated by Harman in 1956, oxidative damage, mainly caused by mitochondria‐generated ROS, leads to limited cellular metabolic function and viability, and compromise both organismic performance and life span (Harman, 1956). Alterations in chromosome number, particularly trisomy 21, have been associated with elevated mitochondrial ROS, oxidative damage, diminished metabolism, and compromised resilience (Busciglio & Yankner, 1995; Coskun et al., 2016; Slonim et al., 2009; Zis et al., 2012) Here, we show that human DS HF and mouse Dp16 MEF display compromised mitochondrial function, oxidative stress, and activated Nrf2 antioxidant response. Furthermore, our results demonstrate that sustained Nrf2 stabilization is vital to maintain cell function in both DS and Dp16. Previous results showed that metabolic recovery in DS cells can be achieved by combined treatment with antioxidants and creatine (Helguera et al., 2013). In agreement with this notion, reduction in mitochondrial ROS by mCAT led to restoration of MMP and normalization of the mitochondrial network in DS cells. Most importantly, mCAT deactivated Nrf2 stabilization, as DS HF treated with mCAT exhibited lower Nrf2 levels similar to NL cells. The localization of mCAT in the mitochondrial matrix appears to be critical to exert its beneficial effect (Schriner, 2005). In that sense, treatment of DS cultures with the mitochondrial‐targeted antioxidants MitoQ and MitoTEMPO had a similar effect, decreasing Nrf2 stabilization (Supporting Information Figure S5D), underscoring the therapeutic potential of optimized antioxidant mitochondrial‐specific interventions.

A similar protective effect was achieved by cCAT expression in PQ stressed cells, but it failed to recover MMP (Figure 1h, i; Supporting Information Figure S2). However, in agreement with previous reports (Bai et al., 1999), cCAT protein displayed a better performance preventing H_2_O_2_ ‐mediated damage (Supporting Information Figure S2C). While PQ‐induced ROS are generated by electrons from respiratory Complex I, inside the mitochondrial matrix, treatment with H_2_O_2_ exposes the cell to exogenous ROS, which explains the differential effects of mCAT and cCAT (Supporting Information Figure S2A).

Oxidative stress in DS has been linked to particular proteins encoded by genes located in Hsa21 that are overexpressed or whose activity is deregulated, namely superoxide dismutase 1 (SOD1), the regulatory kinase Dyrk 1A (DYRK1A), the transcription factor Ets‐2, or the amyloid precursor protein (APP); all of which are directly or indirectly inducers of ROS (Busciglio et al., 2002; Rodríguez‐Sureda et al., 2015; Wolvetang et al., 2003). From a broader perspective, transcriptome studies in DS revealed multiple extra Hsa21 deregulated genes and activated pathways (Bosman et al., 2015). These alterations are associated with a specific transcriptional fingerprint of DS involving mostly genes located outside the supernumerary chromosome, e.g. secondary dysregulations on disomic genes (FitzPatrick, 2005).

Notably, both transcriptional and phenotypic alterations are recapitulated in DS mouse models (Guedj et al., 2016). The Dp16 segmental trisomy carries Hsa21 syntenic fragments, and, unlike the Ts65Dn mouse model, does not include nonsyntenic Hsa21 genes (Li et al., 2007).

In a previous report, in‐silico analysis revealed activation of the Nfr2 pathway in DS astrocytes (Helguera et al., 2013). Here we expand those results and show stabilization and accumulation of Nrf2 protein essential for cell survival in both DS HF and Dp16 MEF. Interestingly, we have observed the stabilization of cytoplasmic Nrf2 in other human aneuploidies (unpublished results) in accord with the notion that gene dosage imbalances result in cellular metabolic stress regardless of the chromosomes involved (Sheltzer et al., 2012). The activation of Nrf2 is consistent with the presence of chronic oxidative stress (Nguyen et al., 2009; Zhang & Hannink, 2003) and emphasizes its key role in activating cellular antioxidant pathways which are critical to curtail oxidative damage. In this regard, mutations in progerin contribute to oxidative damage and accelerated senescence by blocking Nrf2 nuclear translocation and therefore increasing cellular vulnerability (Kubben et al., 2016).

Nrf2 stabilization in DS cells depends on its phosphorylation by PKCδ, which in turn is activated by casp3, as shown by specific inhibitor assays (Niture et al., 2014), demonstrating the increasingly complex role of caspases in physiological and cytoprotective signaling pathways beyond their well‐characterized role in apoptosis (Unsain & Barker, 2015). Alternative and sometimes complementary antioxidant pathways mediated by p53 and FOXO have been described in the context of more stringent oxidative conditions (Gorrini et al., 2013). In the case of DS cells, p53 activation is associated with an oxidative stress‐dependent mitochondrial death pathway (Wolvetang et al., 2003, Helguera et al., 2005), emphasizing the delicate balance between cytoprotective and pro‐death signals that are required to maintain cell and organismal viability. Consistent with this view, we observed significantly higher frequency of p53 translocation events in DS HF only when subjected to PQ treatment (Supporting Information Figure S6C).

Since metabolic alterations observed in DS HF were closely recapitulated in Dp16 MEF, Dp16 appears to be a viable model to investigate the contribution of mitochondrial deficits to DS‐related phenotypic alterations at the organismal level.

In summary, this study highlights the contribution of mitochondrial oxidative stress to cell dysfunction in trisomy 21 and the key role of Nrf2 stabilization in DS cell homeostasis. Our data on Dp16 cells shows the suitability of this DS model to further testing mitochondria‐targeted antioxidant compounds. The fact that mCAT expression is sufficient for the reversal of redox sensitive Nrf2 stabilization highlights its effectiveness as an antioxidant and underscores the relevance of mitochondrial therapies to preserve viability and recover cellular function in DS and possibly other clinical conditions where energy metabolism is compromised.

## 4 EXPERIMENTAL PROCEDURES

### 4.1 Cell culture

MEF were obtained from C57BL6 wild‐type and DP16 (Yu et al., 2010) mice, as described (Xu, 2005). MEF and HEK293 cells were cultured in DMEM (Gibco, Life technologies) supplemented with 10% FBS (Natocord), 1% Glutamax and nonessential amino acids (Gibco, Life technologies), in a 5% CO2 and 37°C atmosphere. HF from four diploid (NL) subjects and four matched subjects with DS were procured from Coriell Institute Cell Repository and cultured according to the provider's specifications (https://catalog.coriell.org/). All the experiments involving animal manipulations were carried out according to the guidelines established by the Institutional Animal Care and Use Committee (IACUC) of the University of California‐Irvine.

### 4.2 Expression vectors

mCAT sequence was provided by Samuel Schriner (Irvine, CA, USA) and subcloned into a lentiviral vector pLV‐eGFP kindly provided by Dr. Pantelis Tsoulfas (Addgene plasmid # 36083). Two sets of forward primers were used to generate both catalase‐carrying plasmids, one including the mitochondrial targeting sequence (MTS) of the original mCAT vector (mCAT) and other excluding the MTS for cytoplasmic expression (cCAT). The mitochondrial‐targeted YFP plasmid was purchased from OriGene and the ORF subcloned into the same pLenti vector (mitoYFP).

### 4.3 shRNA silencing vector

The short hairpin RNA (shRNA) plasmid was constructed in pSilencer 1.0‐U6 vector (Xia et al., 2003) GGGAGGAGCTATTATCCATTC was used as targeting sequence (Supporting Information Figure S7A). The DNA fragments containing U6‐PKD1‐si and U6‐control‐si were inserted into pCAGIG vector in which the green fluorescent protein (GFP)‐cDNA is under the control of chicken actin‐minimal CMV (CAG) promoter the resulting plasmids were referred to as shNrf2‐GFP and shCtrl‐GFP, respectively.

### 4.4 Reagents and antibodies

Paraquat (PQ)(Sigma Aldrich) was diluted from a 0.1 M stock prepared with ultrapure water (Invitrogen). Antibodies for western blotting and immunocytochemistry: rabbit anti‐catalase (#12980), rabbit anti‐VDAC (#4866) and rabbit anti‐Caspase 3 (#9662) from Cell Signaling; rabbit anti‐catalase (sc‐50508), mouse anti‐p53 (sc‐126) and rabbit anti‐Nrf2 (sc‐722) from Santa Cruz; mouse anti‐ATP5α (ab14748) and rabbit anti‐catalase (ab16731) from Abcam; rabbit anti‐phosphoNrf2ser40 (bs‐2013R) from Bioss; mouse anti‐ PKCδ (cat #36520) from Transduction Laboratories; mouse anti‐Tubulin (ATN01) from Cytoeskeleton. Inhibitors: sodium azide from Fisher Scientific; PKCδ (Gö6983), PKCα/β (Gö6976) and Caspase 3 (Z‐DEVD‐FMK) from Calbiochem. Fluorescent Dyes: propidium iodide (PI), 2′,7′‐dichlorodihydrofluorescein diacetate (DCF), tetramethylrhodamine methyl ester perchlorate (TMRM) from Molecular Probes. Mitochondrial targeted antioxidants: MitoQ, 1 mM stock in H_2_O; and MitoTEMPO, 2 mM stock in H_2_O, from Sigma‐Aldrich. Nrf2 activators: Oltipraz (Tocris) 5 mM stock in DMSO; Sulforaphane (Cayman Chemical), 5 mM stock in ethanol.

### 4.5 Mitochondrial network analysis

Quantitative measurement of mitochondrial morphology was performed as described in Zamponi et al., 2018. Briefly, 16‐bit raw pictures were transformed to binary images using an established threshold value, followed by skeletonization of the binary information to obtain an underlying 1 pixel wide structural representation of the mitochondrial network. Considering the number and identity (degree, see Supporting Information Figure S3) of all the nodes within a network, mean degree value (MDV) was calculated for each experimental condition. Given that MDV reflects the overall network connectivity, morphological changes in the mitochondrial networks were estimated by comparing MDV for every condition.

### 4.6 Immunoblotting

Cell homogenates were prepared in RIPA buffer and protein concentration was determined by a Lowry‐based assay (DC Protein Assay Kit, Bio‐Rad). Total protein extracts (30 μg) were mixed with Laemmli buffer (62.5 mM Tris‐HCl, pH 6.8, 2.3 % SDS, 10% glycerol, 5% β‐mercaptoethanol, 0.005% bromophenol blue) and boiled for 10 min. Subsequently, samples were resolved on a 10% SDS‐polyacrylamide gel and electrotransferred to a polyvinylidene fluoride membrane (PVDF, Millipore). After blocking overnight at 4°C in TBS‐Tween 0.05% with 5% nonfat dry milk (TBS‐T 5% DM), membranes were incubated with the appropriate primary antibody diluted 1:1,000 in TBS‐T 5% DM for 2 hr at room temperature, washed 3 × 10 min with TBS‐T, then incubated for 2 hr at room temperature with the corresponding HRP‐conjugated secondary antibody (Jackson Lab) diluted 1:5,000 in TBS‐T 5% DM and washed 3 × 10 min with TBS‐T. Protein levels were detected using ECL mix (WesternBright, Advansta) and radiographic plaques (Fujifilm).

### 4.7 Cell fractionation

Cell fractions were obtained as described (Graham, 2001). Briefly, HEK293 cultures were detached by trypsinization, washed with Phosphate‐Buffered Saline (PBS) and collected by centrifugation at 800 g for 5 min. Cells were then resuspended in 1 ml of ice‐cold RSB Hypo buffer (10 mM NaCl, 1.5 mM MgCl2, 10 mM Tris‐HCl, pH = 7.5), incubated for 10 min in ice and homogenized with 40 strokes in a 1.5 ml glass homogenizer. Immediatly after, homogenized cells were supplemented with 600 μl of ice‐cold 2.5× Homo buffer (525 mM mannitol, 175 mM sucrose, 12.5 mM Tris‐HCl, 2.5 mM EDTA, pH = 7.5) and 800 μl of 1× Homo buffer, and centrifuged at 200 g for 5 min to obtain the nuclear fraction. The post nuclear supernatant was then centrifuged at 9,000 g for 15 min to separate the mitochondria‐rich heavy membrane fraction from the cytosolic fraction. Samples were subsequently resolved by immunoblotting, as previously described.

### 4.8 Immunocytochemistry

HEK293 cells, MEFs and HFs grown on 12 mm glass coverslips (Carolina, Assistant) and treated as indicated in each case, were fixed at 37°C for 20 min with 4% paraformaldehyde‐sucrose solution (PFA). After that, samples were permeabilized with PBS‐Triton 0.1% for 10 min, blocked with PBS‐Horse Serum (HS, Brand) 10% for 60 min and incubated over‐night with the appropriate primary antibody, diluted 1:100 in PBS‐Triton 0.02% with 2% HS. After 6 × 5 min washes with PBS, coverslips were incubated with the corresponding Alexa‐conjugated secondary antibody (rabbit/mouse Alexa 488 or 546), diluted 1:500 in PBS‐Triton 0.02% with 2% HS and washed again 6 × 5 min with PBS. When indicated, samples were also incubated with Alexa‐Phalloidin 546 (1:50 in PBS for 60 min) and DAPI (1:10,000 in PBS for 1 min). Coverslips were mounted using Fluorsave (345789‐Millipore) prior to confocal image acquisition.

### 4.9 Live‐imaging

For live‐cell imaging of human fibroblasts an Olympus IX81 inverted microscope was used, equipped with a Disk Spinning Unit (DSU), epifluorescence illumination (150 W Xenon Lamp) and a microprocessor. Fast image acquisition was achieved with a 60× oil immersion objective and an ORCA AG CCD Hamamatsu camera. Cells were seeded on 25 mm coversilps 3 days before the experiment, to allow proper attachment and habituation. Once ready, coverslips were washed with DMEM without phenol red (DMEMwpr, GIBCO) and incubated for 15 min with the corresponding fluorescent dye (1 μM DCF or 10 nM TMRM) diluted in DMEMwpr. After this time, coverslips were washed again and placed on a imaging ring with DMEMwpr, in the conditions set for the incubator above the microscope platine (37°C and 5% CO_2_). Images were acquired over 20 min per coverslip.

For live imaging experiments with HEK cells, a Zeiss Axiovert 200 epifluorescence microscope was employed, equipped with a 10× dry objective and an ORCA II ER Hamamatsu camera. Cells were seeded on 35 mm plates and treated as indicated in each case, prior to incubation for 15 min with the corresponding dye (2 μM DCF or 10 μM TMRM) diluted in DMEMwpr. After washing the media, plates were placed on an incubator above the microscope platine at 37°C to proceed with the corresponding image acquisition.

### 4.10 Quantitative real‐time PCR (qPCR)

Total cellular RNA was isolated from HF cultures, using TRIzol reagent (Thermo‐Fisher). cDNA was prepared using a cDNA Reverse Transcription kit (Invitrogen). qPCR was performed on a Step One^TM^ Real Time PCR System (Applied Biosystems) by SYBR Green Master Mix reagent detection (Applied Biosystems). The following PCR primers were used: NQO1 rv: GCAAGTCAGGGAAGCCTGGA; NQO1 fw: GGGCAAGTCCATCCCAACTG; HMOX1 rv: GAGGGGCTCTGGTCCTTGGT; HOMX1 fw: GGCCTGGCCTTCTTCACCTT; GAPDH rv: GCCCCAGCGTCAAAGGTGGA; GAPDH fw: ACTGGCGCTGCCAAGGCTGT. All reactions were performed in triplicate, and the experiments were repeated at least three times.

### 4.11 Confocal microscopy

Fixed cells were stained and mounted as explained in the immunocytochemistry section and visualized using an inverted confocal microscope Olympus Fluoview 300.

### 4.12 Electroporation

Human fibroblast cultures were washed, detached and centrifuged as previously described. In each case, 1 × 10^6^ cells were mixed with 2 μg of mitochondrial HyPer (mitoHyPer) along with 2 μg of EV or mCAT plasmids, and transferred to a 1 ml cuvette. Cells were electroporated using a Nucleofector (Lonza), following manufacturer's specifications. Afterwards, electroporated cells were seeded on coverslips, grown for 3 days and fixed with PFA. Mounted samples were analyzed by confocal imaging.

### 4.13 Wound healing assay

HEK cells and human fibroblasts were seeded on a calibrated ibidi confocal μ‐dish (35 mm, glass bottom), and were infected with pLenti‐eYFP and either pLenti‐EV or pLenti‐mCAT . Once confluent, growth media was replaced with DMEM without FBS and cultures were injured with a 0.51 mm‐gauge needle. To discard detached cells, cultures were washed and fresh growth media was added to follow wound assay. Images were acquired with a 10× dry objective every 2 hr for HEK cells, and every 6 hr for human fibroblasts.

### 4.14 Catalase activity assay

Catalase activity was measured as previously described described (Iwase et al., 2013). Briefly, HEK cultures expressing the different plasmids were washed with PBS, detached by trypsinization and centrifuged (800 g – 5 min). Cell pellets were resuspended in PBS and diluted to an even concentration of cell/ml. Afterwards, 100 μl of cell suspention were transferred to a long, 1 cm diameter tube and mixed with 100 μl of PBS‐Triton 1% and 100 μl of H2O2 (30 vol). Reaction took place within 5 min, after what the highness of the bubble column was measured as an indicator of the amount of catalase in the assayed cells.

### 4.15 Cell proliferation assay

To analyze the proliferation activity, infected cells were seeded on 12 mm coverslips and incubated with 1:500 Bromodeoxyuridine (BrdU, Molecular Probes) for 48 hr. Cells were then fixed with ice‐cold Methanol and immunostained against BrdU and counterstained with DAPI. Images were acquired in an epifluorescence microscope with a 10× objective.

### 4.16 Image processing

Images were quantified using Fiji (http://www.fiji.sc). Datasets were analyzed for significant differences with R (http://www.r-project.org).

### 4.17 Statistical analysis

Experiments including three or more groups were analyzed by one‐way ANOVA followed by Tukey's honestly significant difference (HSD) test. Student's *t* test was performed for paired observations. A value of *p* < 0.05 was considered statistically significant. Results were expressed as the mean ± *SD*. Experiments were repeated at least three times, using cultures derived from different NL and DS specimens. Individual experiments were performed in at least triplicate samples.

## CONFLICT OF INTERESTS

The authors declare that they do not have financial or nonfinancial competing interests.

## AUTHOR CONTRIBUTIONS

Experiments were planned and designed by PH, JB, EZ, DC, NZ. Experimental data were generated and collected by EZ, NZ, PQ, GQ. Data analysis and interpretation involved EZ, NZ, DC, SC, PQ, GQ, AL, GP, JB, and PH. Article draft was written by PH, JB, EZ. Critical revisions of the manuscript were performed by AL, GP, KG, SC, DC. Approval of the final version to be published by EZ, NZ, PC, GQ, AL, SC, GP, DC, KG, JB and PH.
