# Supplementary material for: Nrf2 stabilization prevents critical oxidative damage in Down syndrome cells
Source: Aging Cell. 2018 Jul 20;17(5):e12812. doi: 10.1111/acel.12812 (PMC6156351; doi:10.1111/acel.12812)
Supplement: Supplementary file 1 [file ACEL-17-e12812-s001.docx]

**SUPPLEMENTARY MATERIAL**

**­
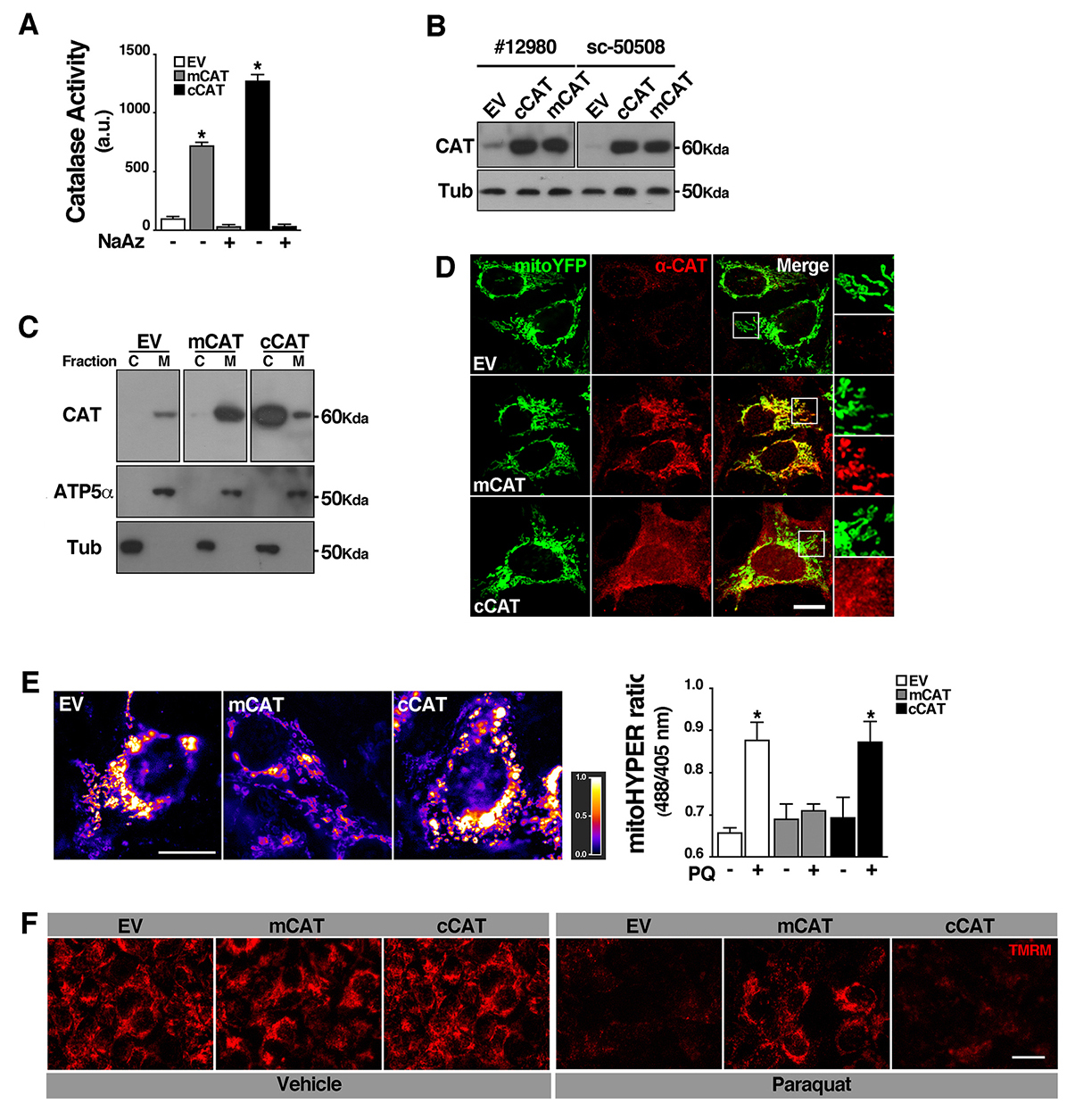
**

**Supplemental Figure 1: Subcellular localization and catalytic activity of mCAT and cCAT.**

**(A)** CAT activity assay was performed on HEK cells expressing EV, mCAT or cCAT constructs and treated for 30 min with 1mM sodium azide (NaAz) to inhibit CAT, or vehicle. Both coded proteins possess catalytic activity**.** ******p* < 0.05. **(B)** Catalase expression was assessed by wb of cells transduced with EV, cCAT or mCAT and detected by two CAT antibodies: #12980 and sc-50508. Tubulin (Tub): loading control. **(C)** Sub-cellular localization of catalase in cells transduced with EV, mCAT or cCAT assessed by wb (see Methods). Fractions: cytoplasmic (c) and membrane-enriched mitochondrial (m). CAT was detected using sc-50508 ab. Tub and ATPase 5 subunit alpha (ATP5ɑ) were used to identify (c) and (m) fractions respectively. (**D)** Immunostaining with anti-CAT antibody (ab16731 - Abcam) shows the localization of CAT (red) in HEK cells co-transfected with EV, mCAT or cCAT and mitoYFP (green). Both coded proteins compartmentalize as expected. Insets: Detail of protein localization is evidenced on single channel images. mCAT channel closely resembles the morphology of the mitochondrial network, while cCAT fluorescence does not. EV has background fluorescence. Scale bar = 10 μm. **Antioxidant and cytoprotective effects of CAT.** CAT constructs prevent oxidative damage HEK cells. **(E)** Quantification of mitochondrial H_2_O_2_ by mitoHYPER radiometric sensor. Cultures expressing EV, mCAT or cCAT were transfected with mitoHYPER for 24 hours and then treated with 5 mM PQ for 48 hours and fixed. ROS concentration was calculated as the ratio between 488 nm and 405 nm fluorescence values for each pixel. Heat bar: Yellow and blue indicate highest and lowest ROS levels respectively. Only mCAT prevented PQ damage.

***** *p* < 0.05. Scale bar = 10 μm. **(F)** Mitochondrial membrane potential (MMP) analyzed with TMRM probe. Cells expressing EV, mCAT or cCAT were treated 48 hours with 5 mM PQ or vehicle; then incubated with TMRM and measured by live-imaging (see Methods). In PQ-treated cultures, there is only a recovery of MMP in cells expressing mCAT. Scale bar = 20 μm.

A: 3 wells (30 cells each) per condition were included for the analysis; E: 20 cells analyzed per each experimental condition; F: 6 wells (four 10X fields each) per condition.


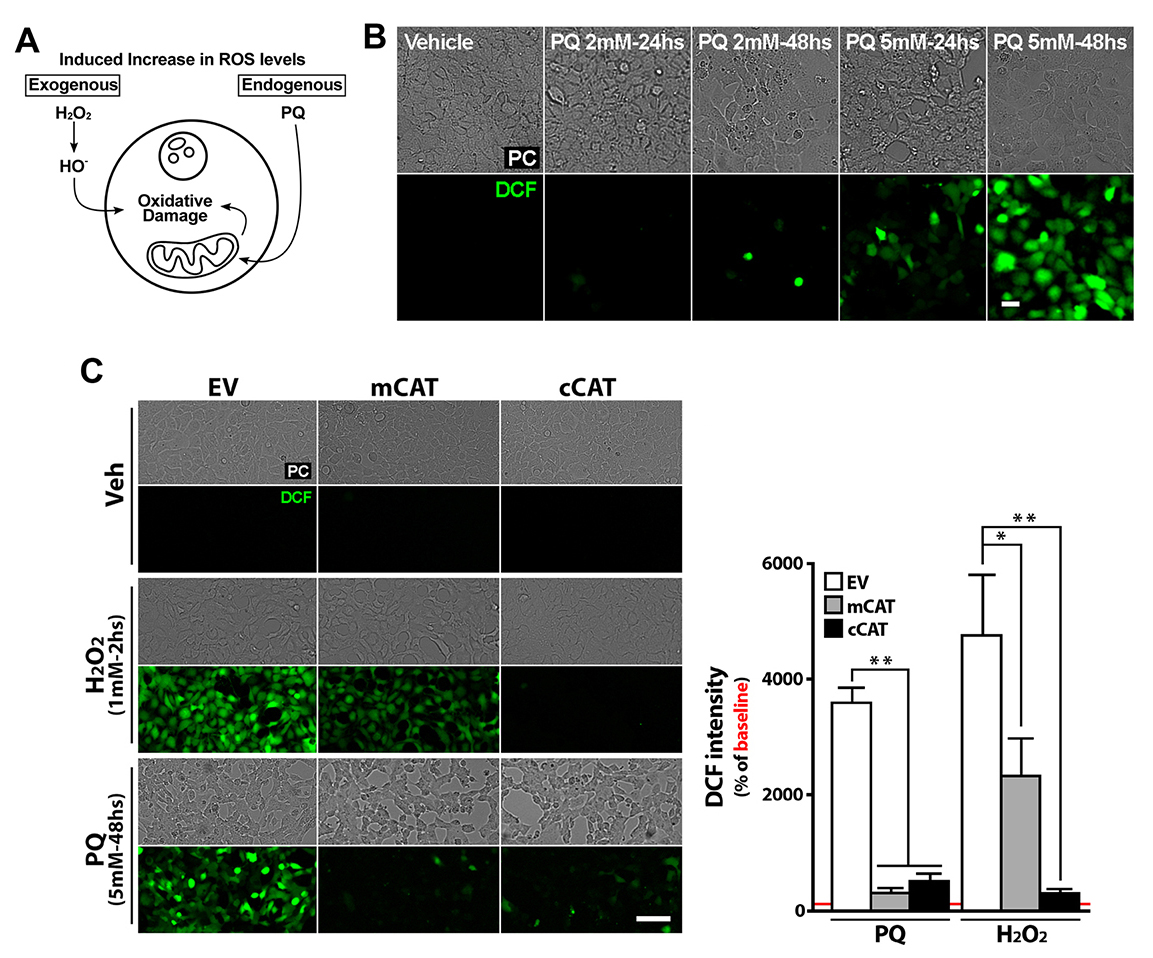


**Supplemental Figure 2: Antioxidant capacity of mCAT and cCAT constructs. (A**) ROS exposure. To evaluate the protective capacity of each catalase construct, HEK cells were exposed to exogenous (H_2_O_2_) or endogenous (paraquat, PQ) ROS. PQ generates ROS by increasing electron leaking from the mitochondrial respiratory chain. **(B)** Adjustment of PQ treatment in HEK cells. Different PQ concentrations (2 and 4 mM) and treatment times (24 hours and 48 hours) were tested in HEK cells. ROS production was visualized with DCF (green, see methods). General culture appearance and morphology was assesed by phase-contrast (PC) imaging. Scale bar = 20 μm. **(C)** Total ROS were measured using DCF in cells infected with EV, mCAT or cCAT and treated for 48 hours with 5 mM PQ, 1 hour with 1 mM H_2_O_2_ or vehicle. Scale bar = 50 μm. Graph: Red line indicates the basal level of ROS in vehicle-treated cultures. There is a marked reduction in the DCF signal in cultures expressing cCAT and mCAT. cCAT is more effective preventing oxidative damage from H_2_O_2,_ while mCAT has a slightly better performance preventing PQ derived ROS. ** p* < 0.05*; ** p* < 0.01*.* C: 6 culture replicates (four 10X fields each) analyzed per condition.


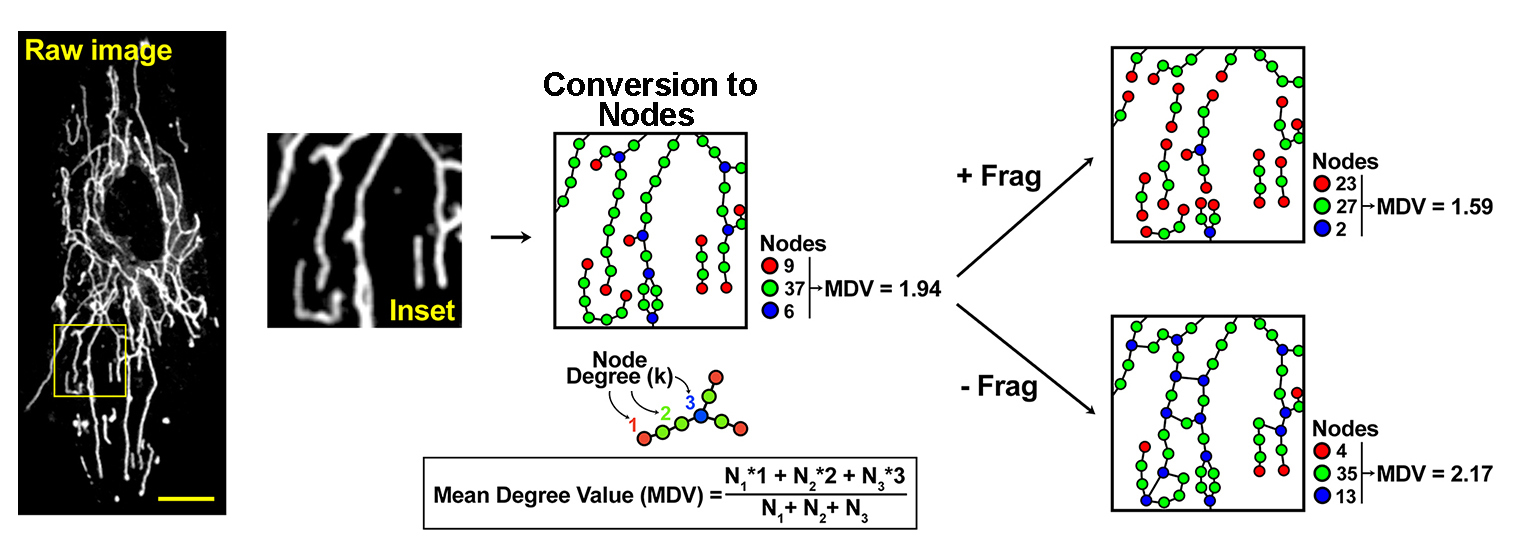


**Supplemental Figure 3: Mitochondrial structural network analysis.** For mitochondrial network analysis in fibroblasts, raw images of mitoYFP (left panel-Inset) were binarized and skeletonized using im2bw and bwmorph MATLAB functions as described (Zamponi et al., 2018) (Conversion to Nodes, central panel). Node Degree (k): Each pixel in the skeletonized mitochondrial network is a node. The degree (k) of each node was determined considering the number of neighboring pixels: degree 1, only one neighbor pixel (red dot), degree 2, two neighboring pixels (green nodes) and degree 3 nodes, three neighboring pixels (blue dot). Higher degree nodes are nearly absent from mitochondrial networks. Mean Degree Value (MDV) The average network node degree (MDV) was obtained applying the detailed equation, and used as a descriptor of network fragmentation. Right Panels: Depending on the relative abundance of higher degree nodes, networks are more fragmented (lower MDV: 1.59) or less fragmented (higher MDV: 2.17).


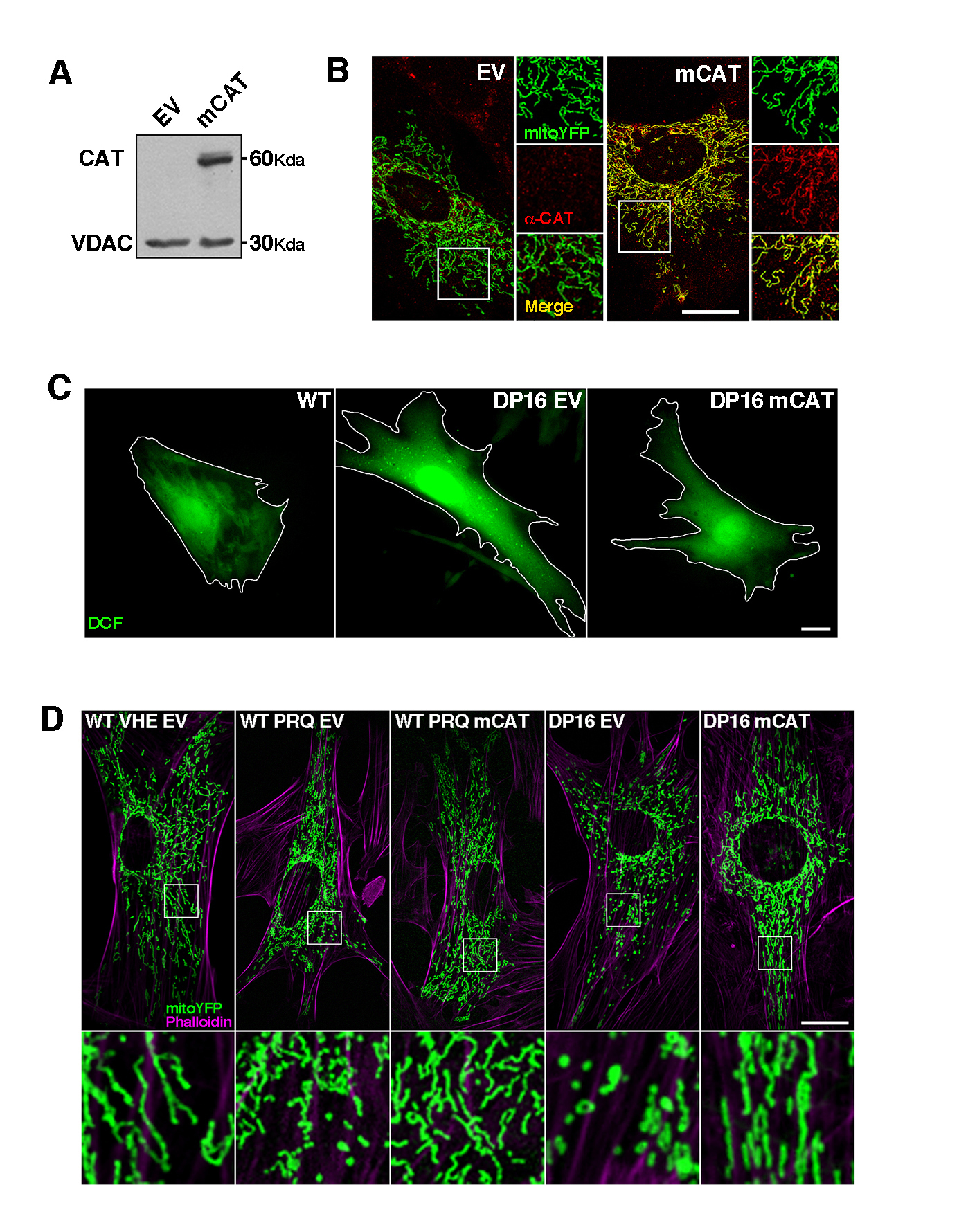


**Supplemental Figure 4: Increased ROS levels and altered mitochondrial network integrity in Dp16 MEF and protection by mCAT. (A)** Western blot analysis of CAT expression in wild type (WT) MEF homogenates transduced with EV or mCAT. Samples were probed against CAT and mitochondrial voltage-dependent anion channel (VDAC). **(B)** Localization of mCAT visualized by confocal microscopy. WT MEF were co-transduced with either EV or mCAT and mitoYFP. Primary antibody: CAT (Ab16731, Abcam), secondary antibody: Alexa Fluor 568. Scale bar = 10 μm. **(C)** Total ROS levels were visualized with DCF as described in Figure 1B and C**.** WT MEF expressing EV (WT), Dp16 MEF expressing EV (Dp16 EV) and Dp16 MEF expressing mCAT (Dp16 mCAT). Scale bar = 10 μm. **(D)** Mitochondrial network in WT and Dp16 MEF co-transduced with mitoYFP and EV or mCAT. WT EV and WT mCAT cultures were treated with 200 mM PQ for 48 hours to induce mitochondrial stress. Alexa-546 phalloidin was used to highlight cell boundaries. Mitochondria images were acquired by confocal microscopy for network analysis. Insets illustrate fragmented mitochondria in both PQ+ WT and Dp16 cells. Scale bar = 10 μm. C and D: at least 20 cells were included per condition for analysis.

**
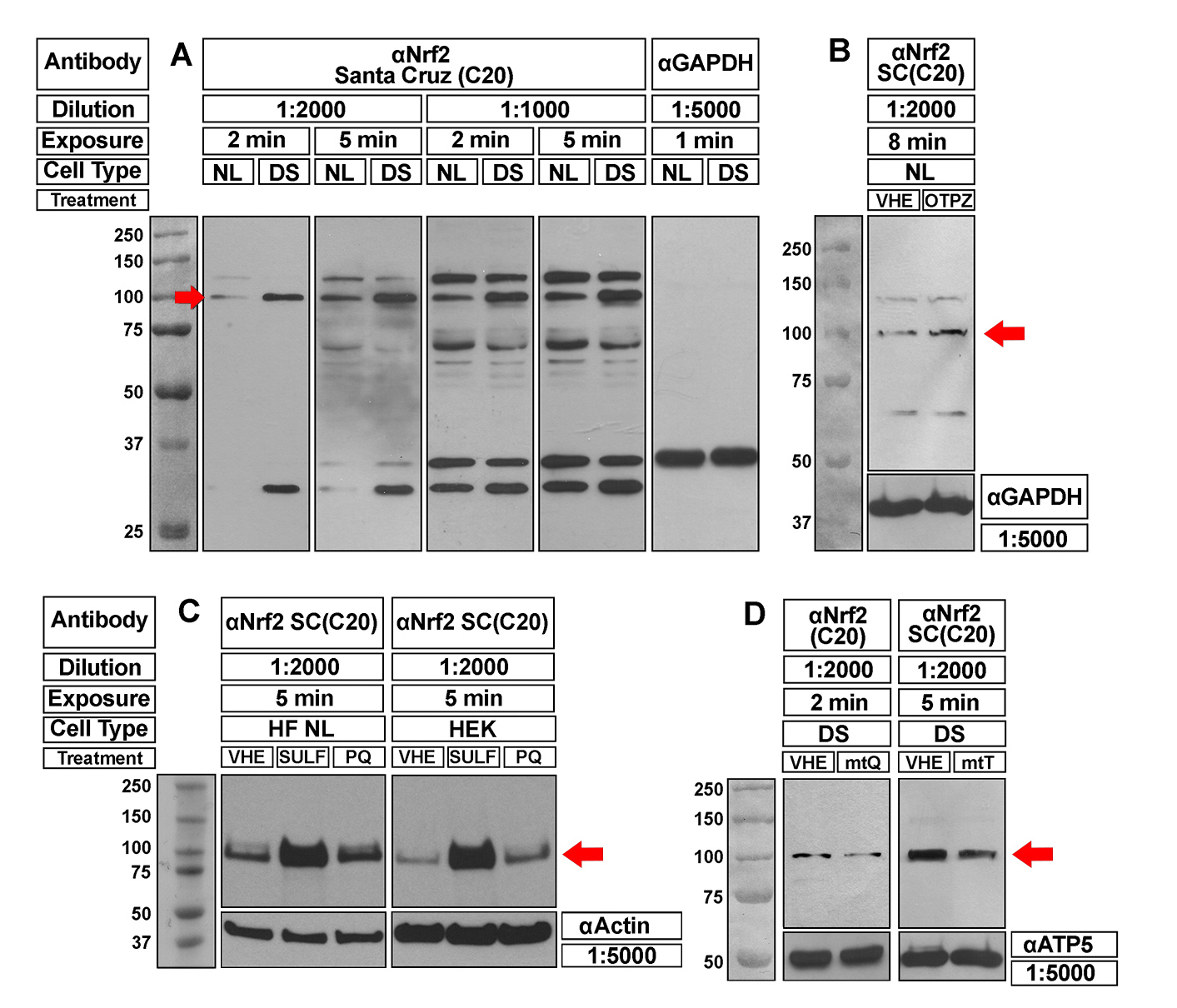
**

**Supplemental Figure 5: Nrf2 detection, induction and destabilization.** (**A**) Calibration of antibody SC-722 (Santa Cruz; aka C-20). Despite its predicted size, Nrf2 protein runs as a 100 kDa band (Lau et al., 2013). A 1:2000 dilution of SC-722 reveals a 100kDa band which is clearly increased in DS samples after a 2 min film exposure (red arrow). A longer exposure (5 min) shows additional bands. A 1:1000 dilution of SC-722 and longer exposures further increased the signal of nonspecific bands. Loading control: GAPDH. **Nrf2 Inducers confirm a 100kD band as native Nrf2 protein.** (**B**) Oltipraz treatment increased Nrf2 protein levels (arrow) in human fibroblasts. (**C**) Sulforaphane (SULF) applied at 5 uM for 4 hours dramatically increased Nrf2 levels (arrow), even more than paraquat (PQ), both in human fibroblasts (left panel) and in HEK cells (right panel). Loading control: actin. **Mitochondrial targeted antioxidant compounds deactivate Nrf2 stabilization**. (**D**) Samples of DS cells treated with either 1μM MitoQ (left panel, mtQ) or 20μM mitoTEMPO (right panel, mtT) displayed reduced levels of stabilized Nrf2 protein (arrow), compared to untreated cells (VHE). Loading control: ATP5.

**
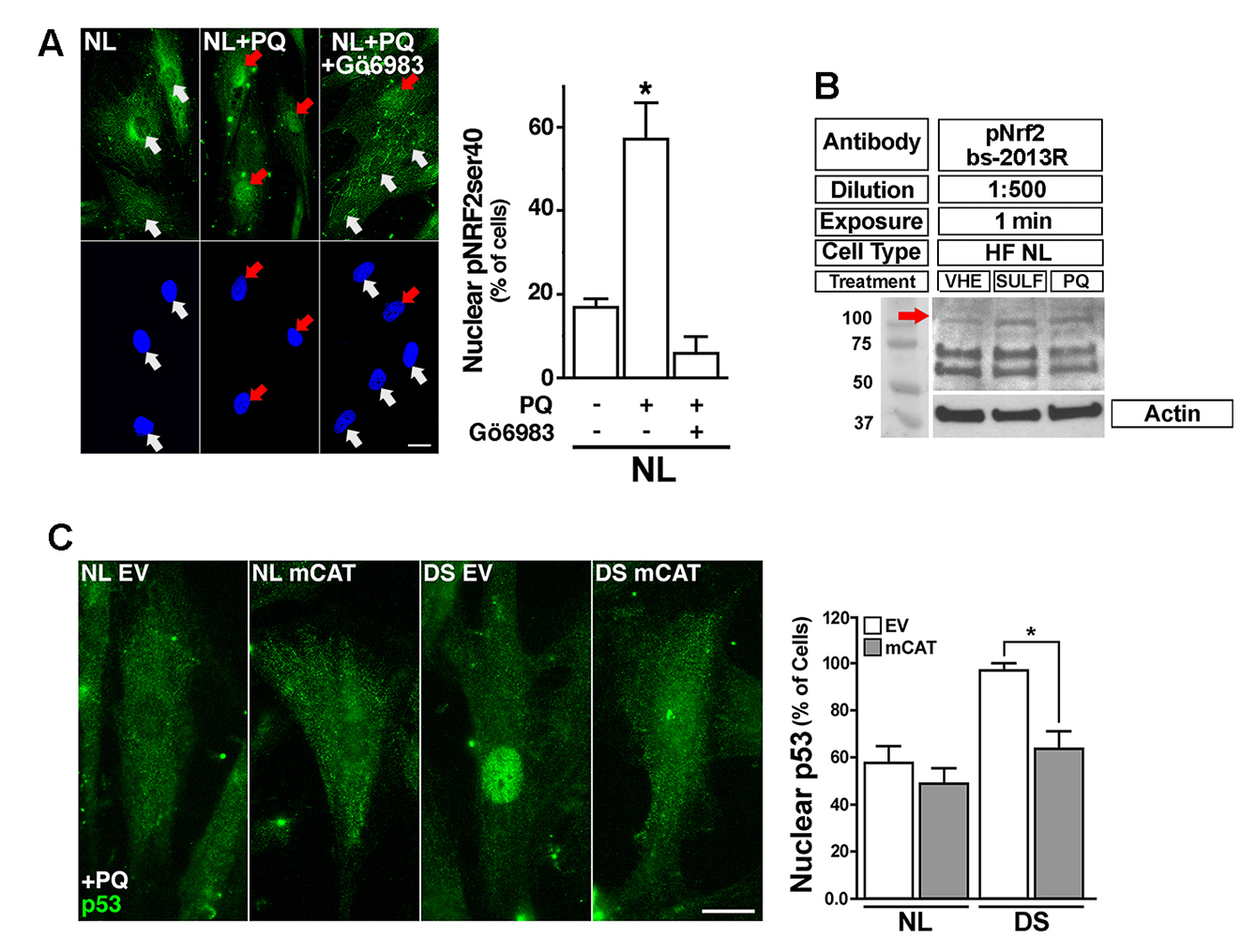
**

**Supplemental Figure 6: Detection of phospho-Nrf2 and p53 in HF. Calibration of Bioss rabbit anti-phospho Nrf2^ser40^ (bs-2013R)**. (**A**) PQ induced pNrf2 nuclear translocation is inhibited by Gö6983 PKCδ inhibitor. Bs-2013R antibody mostly labeled the cytoplasmic compartment in NL cells grown under basal condition (NL, white arrows). Under paraquat driven chronic oxidative conditions (200 μM PQ) there is a dramatic increase in nuclear signal with Bs-2013, consistent with pNrf2 nuclear translocation (NL+PQ, red arrows). Treatment with Gö6983 PKCδ inhibitor (2nM), inhibited paraquat-driven pNrf2 nuclear staining (NL+ PQ + Gö6983). Scale bar = 10 μm, ** p* < 0.05*.*  (**B**) Western blot analysis. Bs-2013R antibody recognizes several bands in human fibroblasts (NL HF). However, a. 100 kDa band (arrow) is the only one that increased intensity in samples from cells treated with the Nrf2 activator sulforaphane, (5μM, 4 hours, SULF) or paraquat (500μM, 12hours, PQ). Actin was used as loading control. **p53 nuclear detection**. (**C**) Increased p53 nuclear translocation in DS cells**.** Under chronic oxidative stress conditions (200 μM PQ), p53 (Green) nuclear translocation is increased in Down syndrome (DS EV) cells, compared to control (NL EV) cultures. mCAT expression reduced p53 nuclear translocation in DS cells (DS mCAT). Scale bar = 10 μm. ** p* < 0.05. A and C: 70-80 cells were included in the analysis per experimental condition.

**
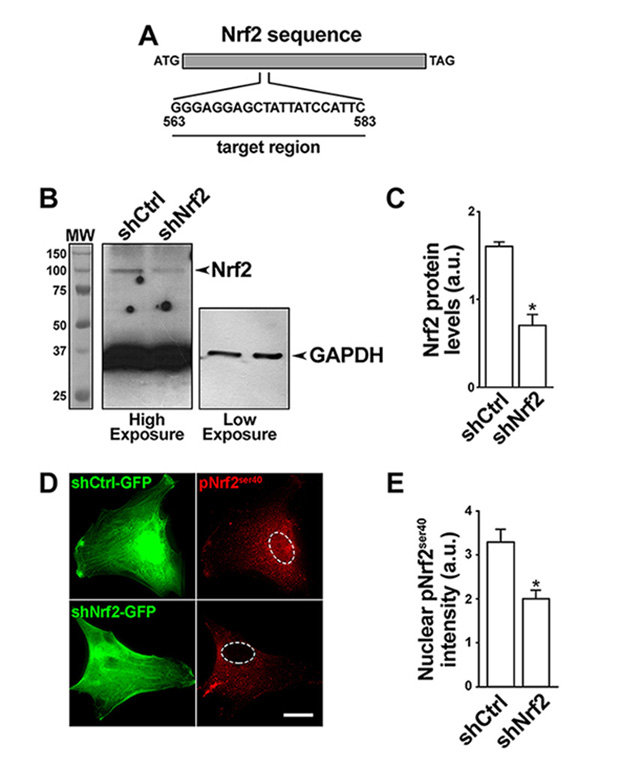
**

**Supplemental Figure 7: Reduced Nrf2 and pNrf2ser40 expression in DS cells treated with a shNrf2 vector. (A)** Nrf2 Immuno-detection specificity was tested by RNA interference (RNAi) using a short hairpin RNA vector (shRNA) that includes the detailed target sequence (See Experimental Procedures). **(B)** WB analysis of DS HF cultures treated either with control (shCtrl) or Nrf2 specific (shNrf2) RNAi vectors, were revealed with SC722 antibody (1:2000 v/v), 5 min exposure (high exposure). GAPDH antibody was used as sample loading control, 1 min exposure (low exposure). **(C)** Densitometric quantification shows that shNrf2 vector significantly reduced native Nrf2 protein levels. * *p* < 0.05. **(D)** Immunofluorescence against active pNRF2ser40 (red) using bs-2013R antibody (Bioss ^TM^) 1:200; performed in DS cultures expressing either shCtrl or shNrf2. Positive sh-plasmid transfection was evidenced by green fluorescence (GFP). **(E)** Nuclear pNRF2ser40 was significantly reduced in shNrf2 treated DS cultures. ** p <* 0.05.


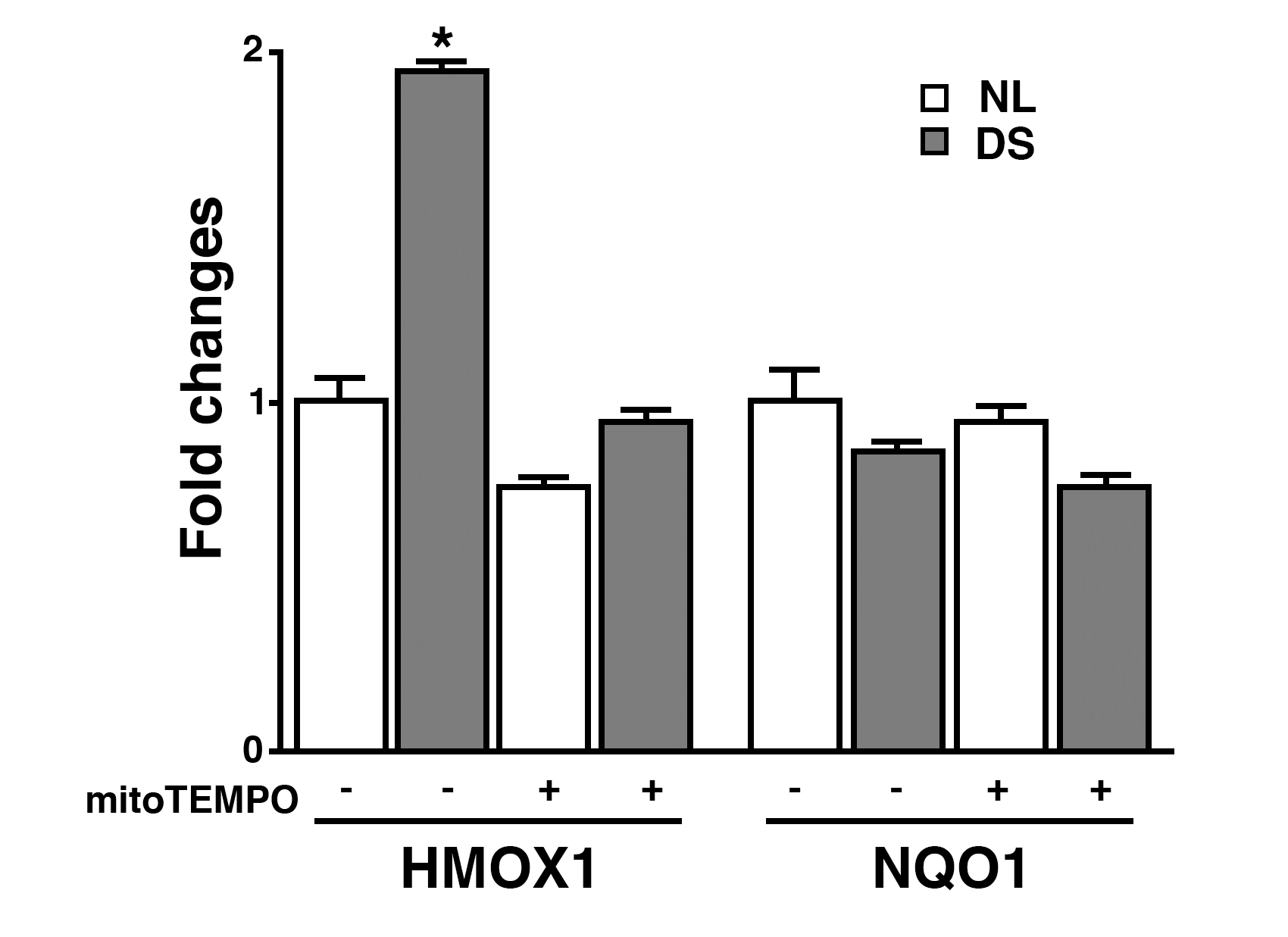


**Supplemental Figure 8: Regulation of Nrf2-induced antioxidant genes in DS HF.** HMOX1 and NQO1 transcription levels are regulated by Nrf2 and share the same Nrf2 DNA consensus sequence (Wang et al., 2007; Zucker et al., 2016). NL HFs (NL) and DS HFs (DS), treated either with 20μM mitoTEMPO antioxidant (+) or vehicle (-), where assayed for transcription levels of HMOX1 and NQO1 genes by qPCR. DS cells exhibited significantly higher expression of HMOX1 and, as expected, antioxidant mitoTEMPO lowered HMOX1 transcript level. Consistent with recent reports, NQO1 transcripts were not increased in DS HFs (Liu et al., 2017). * *p* < 0.02.

REFERENCES

Liu Y., Borel C., Li L., Muller T., Williams E. G., Germain P. L., et al. (2017). Systematic proteome and proteostasis profiling in human Trisomy 21 fibroblast cells. *Nat. Commun.* 8:1212. 10.1038/s41467-017-01422-6

Wang X, Tomso DJ, Chorley BN, Cho HY, Cheung VG, Kleeberger SR, Bell DA (2007) Identification of polymorphic antioxidant response elements in the human genome. *Hum. Mol. Genet.*  16:1188–1200.

Zamponi N, Zamponi E, Cannas SA, Billoni O V, Helguera PR & Chialvo DR (2018) Mitochondrial network complexity emerges from fission/fusion dynamics. *Sci. Rep.* 8, 363. Available at: https://doi.org/10.1038/s41598-017-18351-5.

Zucker SN, Fink EE, Bagati A, Mannava S, Bianchi-Smiraglia A, Bogner PN, Wawrzyniak JA, Foley C, Leonova KI, Grimm MJ, Moparthy K, Ionov Y, Wang J, et al. (2014) Nrf2 amplifies oxidative stress via induction of Klf9. *Mol Cell.* 53:916–28.
